# Cost-Effectiveness Analysis of Hospitalization and Home-Based Care Strategies for People Living with HIV/AIDS: The Case of Zimbabwe

**DOI:** 10.1155/2014/836439

**Published:** 2014-09-28

**Authors:** Senelani D. Hove-Musekwa, Farai Nyabadza, Hermane Mambili-Mamboundou, Christinah Chiyaka, Zindoga Mukandavire

**Affiliations:** ^1^Department of Applied Mathematics, National University of Science and Technology, P.O. Box AC 939, Ascot, Bulawayo, Zimbabwe; ^2^Department of Mathematics, University of Stellenbosch, Private Bag X1, Matieland 7602, South Africa; ^3^School of Mathematics Statistics and Computer Science, University of KwaZulu Natal, Durban Road, Pietermaritzburg 3201, South Africa; ^4^School of Social and Community Medicine, University of Bristol, Canynge Hall, Whatley Road, Bristol BS8 2PS, UK; ^5^London School of Tropical Hygiene and Medicine, Department of Global Health & Development, Keppel Street, Bloomsbury, London WC1E 7HT, UK

## Abstract

The model of care of people living with HIV/AIDS (PLWHA) has shifted from hospital care to community home-based care (CHBC) because of shortage of space in hospitals and lack of resources. We evaluate the costs and benefits of home-based care and other HIV/AIDS intervention strategies in Zimbabwe, using an interdisciplinary approach which weaves together the techniques of an epidemic transmission model and economic evaluation concepts. The intervention strategies considered are voluntary counselling and testing (VCT), VCT combined with hospitalization (H), VCT combined with CHBC, and all the interventions implemented concurrently. The results of the study indicate that implementing all the strategies concurrently is the most cost-effective, a result which also agrees with the epidemiological model. Our results also show that the effectiveness of a strategy in the epidemiological model does not necessarily imply cost-effectiveness of the strategy and behaviour change, modelled by the parameters *p* and *m*, that accompanied the strategies, influencing both the cost-effectiveness of an intervention strategy and dynamics of the epidemic. This study shows that interdisciplinary collaborations can help in improving the accuracy of predictions of the course and cost of the epidemic and help policy makers in implementing the correct strategies.

## 1. Introduction

The HIV/AIDS epidemic has become the world's most destructive epidemics recorded in history. The number of people living with HIV worldwide was estimated to be 33.4 million [1]. Sub-Sahara Africa accounts for 22.4 million [2] and remains the region most heavily affected by HIV with an adult prevalence rate of over 15% in some countries [3]. The majority of those infected in sub-Saharan Africa are unaware of their status [4].

The first reported case of AIDS in Zimbabwe occurred in 1985. By early 1990s around 10% of the adult population was thought to be infected with HIV. This figure rose dramatically in the first half of the 1990s, peaking and stabilizing at 29% between 1995 and 1997 [1]. Although survey results do indeed indicate a fall in Zimbabwe's adult HIV prevalence, a rise in the number of people dying from AIDS is thought to have played a role in the decline, as well as an increase in the number of people (HIV positive or otherwise) who might have migrated to other countries. Nonetheless, there is evidence that Zimbabwe's HIV prevalence has genuinely fallen and that changes in sexual behaviour have played a role in achieving this [5]. The prevalence almost halved to 16% in 2007 from 29% in 1997 [1]. It is thought that an increased awareness of HIV and AIDS has influenced these changes. Condom use has increased, a number of young people are delaying first sex, and many people have reduced their number of sexual partners [6]. In many cases, people changed their behaviour after witnessing the effects of the epidemic first hand, through the death of friends or relatives, and this was helped by HIV/AIDS prevention programmes like home-based care. Latest data shows that Zimbabwe HIV prevalence has continued to drop rapidly. The data shows that overall HIV prevalence among pregnant women who attended antenatal clinics decreased from 23% in 2001 to 11% towards the end of 2009 [7]. The prevalence in the general population was found to be 13.2% in 2008 [8].

Economic epidemiology, which is a field at the intersection of epidemiology and economics, can influence HIV/AIDS policy decisions. Its main aim is to incorporate principles of individual behaviour, incentives for healthy behaviour, resource optimization, resource allocation, and simple economics into epidemiological models and conversely the dynamics of infectious diseases into health economics. Economic epidemiological modelling can provide a key input in decision making since it has systematic and quantifiable assessments for different intervention strategies. Economic evaluation concepts need to be employed to ensure that any new resources for the epidemic will have the maximum possible effect on the epidemic. One of the concepts involved is cost information which is a measure of both cost and cost-effectiveness. For efficient allocation of HIV/AIDS resources, decision makers must understand better the impact and cost-effectiveness of HIV/AIDS prevention and treatment programs. Therefore, cost-effectiveness should be considered when designing strategies for prevention, care, and support for HIV/AIDS [9]. There is very little compiled information on the relative cost and likely impact of each intervention in different settings either individually or in combination. While there have been some reviews of HIV/AIDS prevention strategies with cost-effectiveness analyses (see, for instance, [9–12]) very few have combined mathematical epidemiology and economic modelling in assessing the cost-effectiveness of prevention and treatment strategies. Many mathematical models that incorporate a number of strategies to combat the epidemic have been developed [4, 13–15] and the references cited therein. However, resources to be used to implement and maintain these strategies must be measured, valued, and costed. It is also important to know the health benefits, like number of infections averted, number of deaths averted, life years gained, and the cost of achieving these benefits.

Strategies developed to control the spread of HIV include different forms of behavioural change and communication, voluntary counselling and testing, promotion of male and female condoms, harm reduction strategies among drug users [4, 13–15], and the references cited therein. In poor resource settings, the model of care of people living with HIV/AIDS (PLWHA) has shifted from hospital care to community home-based care (CHBC) because of shortage of space in hospitals and lack of resources. Long periods of hospitalization are not an option irrespective of a patient's needs. Limited resources always present a huge challenge in their allocation. There is therefore a huge tradeoff between costs and patients' needs. Individuals released from hospital are put in CHBC programs for the continuation of care. These programs are run through resources that have to be allocated with other care programs and social needs in mind. The question that arises is how best can we allocate the few available resources and at the same time derive maximum benefit from each of the prevention and care programs? This question can only be answered by a combination of a mathematical model that incorporates prevention and care programs with economic modelling on resource allocation.

The paper follows the work presented in [16]. The model and its equations are given in Appendix B while the description of state variables and parameters is given in Appendix A. This paper ensures that economic epidemiology principles take the centre stage in analysing the cost-effectiveness of the prevention strategies and treatment. The cost-effectiveness analysis will thus be based on the epidemiological model in [16]. While some strategies are very cost-effective in the short term, they may be costly and unsustainable if implemented over a long period of time. Other strategies are costly in the short term; their long-term benefits may reduce the costs due to their impact in controlling the epidemic. Therefore, the aim of the paper is to compare the costs and benefits of the different types of strategies with particular reference to the HIV/AIDS epidemic in Zimbabwe.

We use this epidemiological model to track the economic costs which account for the economic consequences of the epidemic. The model assesses the impact of four strategies: voluntary counselling and testing (VCT), VCT combined with hospitalisation, VCT combined with CHBC, and a combination of the three strategies. The results presented in [16] showed that a combination of all the intervention strategies gives the best result followed by the VCT and hospitalisation, VCT and CHBC, and VCT alone. The thrust of this paper is to compare the cost-effectiveness of these strategies and verify whether their impact in the given order is cost-effective. The total health benefits and costs of the three strategies are analysed to determine how combinations of various factors affect the cost-effectiveness of the strategies. This helps to quantify years of perfect health gained, measured as quality-adjusted life years (QALYS), and the burden of the disease analysis on the human population measured in terms of disability-adjusted life years (DALYs) lost, that is, years of perfect health lost [17]. To determine whether the added effectiveness in the different interventions is worth the added cost, cost-effectiveness ratio will be calculated in the form of the incremental cost-effectiveness ratio (ICER).

## 2. Epidemiological Measures

### 2.1. The Basic Reproductive Number

In epidemiological modelling, the main goal is to deduce conditions necessary and/or sufficient for disease elimination and/or eradication. To achieve this we use the basic reproduction number *R*_0_. It has a threshold value of 1, below which the generation of secondary cases is insufficient to maintain the infection within the human community. If *R*_0_ > 1, each infected individual produces more than one new infected individual and hence the disease is able to invade the susceptible population [18]. In the presence of an intervention strategy, we have an effective reproduction number, *R*_*e*_, which has to be compared with *R*_0_. From the epidemiological model in [16], *R*_*e*_ is a sum of four terms representing the contributions of the unidentified infective individuals, the screened and identified infected individuals, the AIDS individuals, and PLWHA under community home-based care. The different interventions are represented by different effective reproduction numbers which are as follows: no interventions, *R*_0_, screening and counselling only, *R*_*es*_, screening and counselling coupled with hospitalization, *R*_*esh*_, screening and counselling coupled with CHBC, *R*_*esh*_*b*__, and finally screening and counselling, hospitalization, and home-based care represented by *R*_*e*_ and this is shown in Appendix C. It was shown that

(1) $$\begin{array}{c} R_{e}<R_{esh}<R_{esh_{b}}<R_{es}<R_{0}, \end{array}$$

showing that the most effective intervention is using a combination of all the suggested interventions followed by voluntary counselling and screening accompanied by hospitalization and voluntary counselling and screening alone is the least effective intervention.

### 2.2. Incidence and Mortality

Incidence and mortality rates are used to calculate the DALYS. In [16], the model was fitted to the current prevalence data on Zimbabwe from the UNAIDS/WHO reports and epidemiological fact sheets [23, 24] and, using the least squares curve fitting in MATLAB, the lower and upper bounds of specific parameters to be estimated were specified and they are given in Table 9. Population estimates for 1990 were used to establish the initial conditions. Zimbabwe's population was estimated to be 10.156 million in 1990, with a life expectancy of 59 years by the United States Bureau of the Census [25]. The estimated adult HIV prevalence was from the age group 15–49 and this was used to estimate the initial population of adults aged between 15 and 49 in 1990. The following initial conditions

(2) (S(0),I(0),Is(0),A(0),H(0),Hb(0))  =(4519960,550000,50000,100000,3640,36400),

corresponding to an initial prevalence of 14%, were used. The same data was used to estimate the annual number of new infections generated and the mortality rate. The incidence and mortality were evaluated from the following expressions:

(3) Incidence=βe−m(δ1A+δ2H+δ3Hb) ×(I+(1−p)(η1Is+η2A+η3(1−ϕ)Hb)N)S,mortality=(δ1A+δ2H+δ3Hb)N.

It was shown that if more identified infective individuals join the CHBC, that is, increasing *ρ*_1_, the rate of seeking care and treatment from CHBC, the prevalence of the disease decreases such that doubling *ρ*_1_ reduces the prevalence by 1.3%, from 8.2% to 6.9%, and a four-time increase in *ρ*_1_ will reduce the prevalence by 3%. However, increasing the rate of seeking care and treatment from CHBC of discharged AIDS individuals from the hospital, *ρ*_2_, increases the prevalence of infection implying that recruitment of discharged individuals from hospitals into CHBC has a negative impact if the individuals remain a potential source of infection. This means that reducing the infectivity of individuals in CHBC should remain the main focus of the care program for effective disease control. It was also shown that if more people withdraw from risky sexual behaviour and the effectiveness of CHBC is increased, that is, increasing *p* and *ϕ*, respectively, the prevalence of HIV decreases.

### 2.3. Infections and Deaths Averted

Since intervention strategies are designed to change the course of an epidemic, that is, reducing the number of new infections and preventing deaths, the number of primary infected individuals before any intervention was estimated to be 4.9819 × 10^4^ per year. The annual number of deaths on average when there is no intervention was 1.3755 × 10^5^. These estimates were done by using a fourth order Runge-Kutta scheme in MATLAB. These are used to estimate the burden of disease and help in the public health planning policy.

### 2.4. Measuring the Economic Impact of HIV/AIDS

The economic impact is assessed in terms of cost and cost-effectiveness of the different intervention strategies. This involves cost measures for each strategy and the different economic evaluation methods which will be discussed in the next sections. Since voluntary counselling and screening is the common strategy which is in place, in our cost-effectiveness analysis, we take VCT as the existing strategy in addition to the “no intervention” basis.

### 2.5. Cost Measurement

In measuring costs, we identify the resources to be used, quantify them, and place a monetary value on them. We calculate and compare the costs of interventions per health outcome achieved to meet social objectives, that is, maximization of total population health. No monetary value is assigned to outcomes but rather results are presented in the form of cost per health outcome, like costs per HIV infection averted and cost per life saved. The costs include direct, indirect, and intangible costs. Costs of all resource inputs and existing infrastructure are indirect and intangible costs including the pain of suffering (morbidity) to the patients and their families which are difficult to measure since they do not have a market value. Morbidity and mortality (death) costs are captured in the calculation of the cost-effectiveness ratio. Since costs fluctuate with time, in economic analysis there is discounting of the costs for the particular period and this discount rate is normally between 3% and 5%. According to Golg et al. [26], all future costs and effectiveness values must be discounted into their net present value. Although standards vary from country to country internationally, many studies use a 5% discount rate [21]. We will thus use the same discount rate for our analysis.

We assume that one way of showing change of behaviour, which is represented in the epidemiological model by the proportion of individuals who withdraw from risky sexual behaviour, *p*, is by the use of condoms. A proportion *τ*_*i*_, *i* = 1,2, 3,4, 5 from the sexually active population of susceptible, unidentified infective, screened infective, symptomatic individuals with AIDS and those in CHBC, respectively, are assumed to use condoms. Thus, the cost of condoms, *C*_*c*_, is proportional to the total number of sexually active individuals at any given time. The cost of screening, *C*_*s*_, is associated with the fraction of infected individuals who choose to be screened and counselled and the proportion of the individuals with clinical AIDS who will be screened from both the unidentified infective individuals *I* and the screened and counselled *I*_*s*_ moving into the AIDS class. Screening is performed in primary care clinics and nongovernmental organisations (NGOs) centers or at CHBC centers for anyone who requires the service. The test costs include all related expenditures for testing. We assumed that individuals can go for screening twice a year at cost of $10 per test [19]. The costs, *C*_*AH*_, of treating those who would have developed clinical AIDS and are hospitalized are determined by the proportion of the individuals who will be hospitalized having developed clinical symptoms of AIDS. These costs include the overhead costs of medicines for treating opportunistic infections like tuberculosis, cost of antiretroviral therapy, cost of hospital bed, and palliative care. Since we do not have any data on the management of the AIDS patients admitted in the hospitals and those who seek treatment from home-based care institutions, we make estimates based on costs from literature. Costs for home-based care, supply of structured ARV treatment, and other medical treatments are *C*_*H*_*b*__. The costs of running home-based institutions and other related administrative issues, training peer educators, and printing booklets and other related materials are taken to be *C*_*R*_. All the costs are annual cost to prevent and/or treat an individual in a particular disease stage. Therefore, the total cost rate function, *C*(*t*), (in US $ per unit time) at time *t*, is given by

(4) C(t)=Cc[τ1S(t)+τ2I(t)+τ3Is(t)+τ4A(t)+τ5Hb] +Cs[σI(t)+γ1I(t)+γ2Is(t)] +CAHA(t)+CHbHb(t)+CR.

The total costs, *C*_TC_, are the cumulative costs spread over 19 years, from 1990 to 2009. They are calculated by summing up all the costs for the different intervention strategies. The total discounted economic costs are calculated using a discount rate of 5% which is very consistent with contemporary standards in developing countries. The total discounted economic costs per person, direct costs of treatment, and prevention costs over the period of 19 years are given by

(5) $$\begin{array}{c} C_{\text{TC}}=\overset{19}{\underset{0}{\int }}C\left(t\right)e^{-rt}dt, \end{array}$$

where *r* is the discount rate. The average annual costs can be calculated from cumulative costs for the entire period.

## 3. Economic Measures

One of the health outcomes (health benefits) is the quality-adjusted life years (QALYs). It is a reflection of “the valuation that a year of life with HIV infection is less desirable than a year of life without HIV infection and a year of life with asymptomatic HIV infection is more desirable than a year of life with symptomatic HIV infection” [22]. Therefore, individuals who benefit from an intervention program that moves them from a lesser health state to an improved health state for some period of time will have gained and enjoyed better health. Health benefits are measured by the duration of life years gained as a result of an intervention. Using the epidemiological model in Appendix B, the summation method was used to calculate the QALYs using the appropriate weights from the literature [22].

To make a decision on which intervention to choose, we first calculate a cost-effectiveness ratio (CER) in the form of incremental cost-effectiveness ratio (ICER). The effectiveness of an intervention is measured in terms of QALYs or just infections averted. This depends on the epidemiological and demographical factors and the capacity to implement the strategy [4].

The ICER is more relevant when there is more than one intervention strategy to be considered because it helps policy makers to decide whether to remain with an existing intervention or adopt a new intervention. The interventions considered in this paper depend on VCT and therefore we consider VCT combined with hospitalization and VCT combined with community home-based care. Finally we analyse the combined interventions, that is, all the three applied together. To determine the cost-effectiveness of each suggested strategy, we take VCT as the existing intervention and then calculate the incremental cost-effective ratio (ICER) since all the other proposed strategies rely on VCT. At this point, it is understood that a number of people are aware of the VCT programs and therefore we use the ICER as our measure to determine cost-effectiveness of the interventions.

We calculate the total discounted QALYs lived by the population in a particular disease stage using the following equation:

(6) $$\begin{array}{c} Q\left(t\right)=\overset{19}{\underset{0}{\int }}\overset{j=3}{\underset{j=0}{\sum }}\;\overset{i=5}{\underset{i=0}{\sum }}q_{i}V_{ij}\left(t\right)e^{-rt}dt, \end{array}$$

where *q*_*i*_ are the QALY adjustment for a year of life in a particular disease stage *V*_*i*_ and number of interventions *j*. No intervention, VCT only, VCT and hospitalization, VCT and CHBC, and all interventions are represented by *j* = 0,1, 2,3, respectively. This means that if *j* = 1, it implies VCT only, if *j* = 2, that means either VCT and CHBC or VCT and hospitalization, and finally if *j* = 3, we have VCT, hospitalization, and CHBC. The values of *q*_*i*_ vary from 0.171 in the hospitalized class to 1 (representing perfect health in the susceptible class). The total discounted costs and QALYs gained are determined for single or/and combined interventions as well as for no intervention at all. The difference in the total discounted costs and QALYs accrued in the population with and without interventions is also considered.

Input data was obtained from the numerical simulations of the epidemiological model which was fitted to the HIV prevalence data for Zimbabwe [16]. The economic parameter values are given in Table 1 and the epidemiological model parameters are in Table 9.

## 4. Results and Discussion

**Figure 1:**
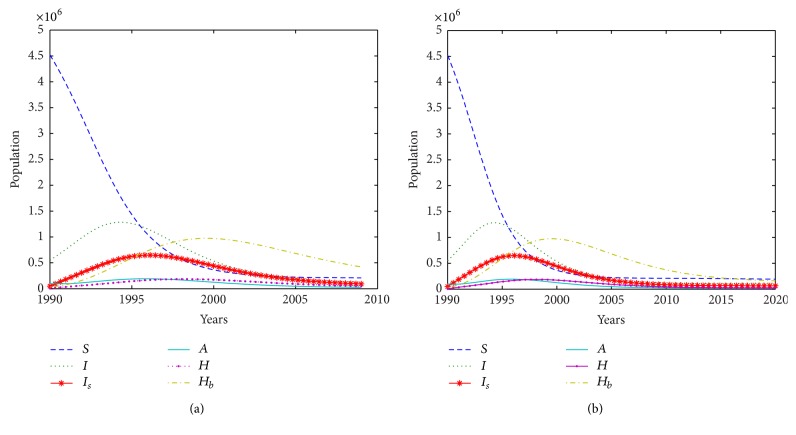
Trend of the epidemic over (a) 19 years and (b) 30 years.

The general dynamics of the epidemic based on the information in Table 9 is shown in Figure 1.

Figure 1 tracks the changes in the populations in each compartment. For example, the change in the population of individuals taken into CHBC, hospitalized, with AIDS, and those who are screened can be tracked annually for the purposes of estimating the burden of disease and public health planning. In Figure 1(a) the epidemic remains within the population at low levels with most of the infected turning to home-based care facilities. The number of the identified and screened infective individuals increases and then declines as more of them join the community home-based care. However, in the long run, as shown in Figure 1(b), the population will have more susceptible individuals remaining compared to the other groups of the population. It is also encouraging to note that few individuals progress to full blown AIDS.

**Figure 2:**
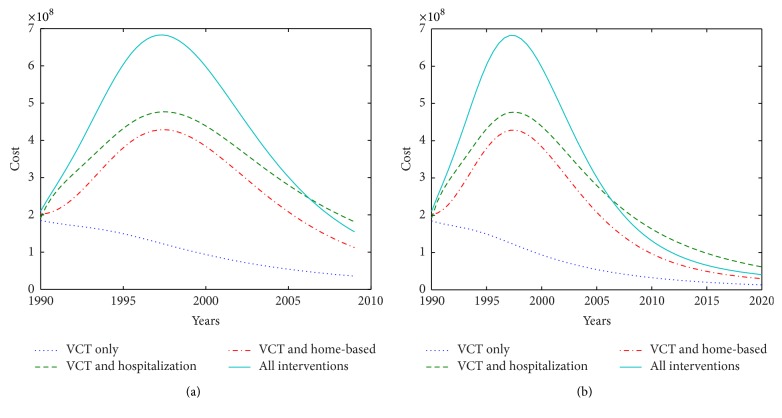
Trend of cost for each strategy per year for (a) 19 years and (b) 30 years.

The costs of each strategy can also be tracked over the number of years once the number of individuals in each epidemiological class can be tracked annually.  Figure 2(b) shows the change in the costs for each strategy over the 19 years (Figure 2(a)) and 30 years (Figure 2(b)).  Figure 2 shows that costs are saved as a result of infections being averted and this is indicated by the costs decreasing with time annually. In the long run the cost of implementing the combined strategies is cheaper than VCT and hospitalization.

To evaluate the number of infections and deaths averted, we consider two scenarios; firstly we consider a situation where there is no behavior change, that is, *m* = 0, and secondly we consider a situation where there is behavior change, that is, *m* ≠ 0. We assume that mortality due to AIDS drives behaviour change as people endeavor to avoid getting infected. The number of infections and deaths averted is calculated as the difference between the infections or deaths when there is no intervention and when there is an intervention singly or in combination. For the given parameter values in Table 9, when there is no change of behaviour, the infections (49 632) and deaths (1.76 million) are more than when there is behaviour change, so that they are 42 849 and 1.38 million, respectively, per year with no intervention. This shows that mortality due to AIDS influences people's behaviour resulting in the reduction of transmission. Reduced transmission leads to less people being infected and dying from the disease.

All the interventions, individually or in combination, reduce the number of deaths due to the disease. We note that the greatest number of infections and deaths averted is from the implementation of all intervention strategies and the least is from an intervention with VCT only. The implementation of VCT and CHBC prevents more infections and deaths than VCT and hospitalization. Therefore, the strategy with VCT and CHBC is both cheaper and effective in reducing the number of infections compared to the strategy with VCT and hospitalization as can be seen by the costs of infections or deaths averted. Depending on the availability of funds for the intervention strategies, implementing all the suggested strategies will depend on their cost-effectiveness. These results are used in the determination of the cost-effectiveness of the interventions. This is done by finding the incremental cost-effective ratio, ICER.

**Figure 3:**
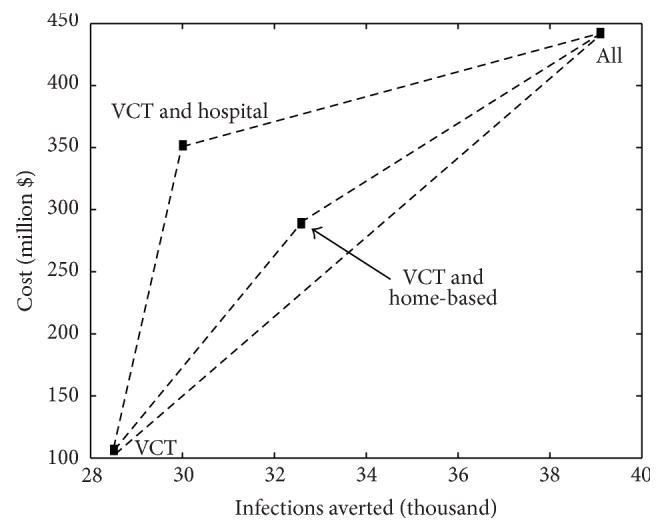
The cost and cost-effectiveness of the three strategies with VCT as the existing strategy.

Taking VCT as the existing intervention, the ICERs for the VCT plus hospitalization, VCT plus CHBC, and ALL are calculated and shown in Table 2. This helps us to determine the cost-effectiveness of each suggested strategy by determining the increase in cost for each strategy used instead of VCT only. The total discounted costs and infections averted per year for each strategy are presented as a point on the cost-effectiveness plane in Figure 3. From the ICER analysis in Table 3 and Figure 3, all the programs are potentially efficient as they all increase the infections averted but at a higher cost. A decision to choose a strategy which represents good value for money is often difficult to make but the cost-effectiveness graph in Figure 3 becomes handy. The ICER represents the gradient of the line connecting the program outcome to VCT on the cost-effectiveness graph. The program which has the lowest gradient or with flatter slope is the most cost-effective. This implies that implementing all the intervention strategies will be the most cost-effective strategy since the incremental cost, $31 798, per infection averted, is the lowest followed by VCT and CHBC ($45919 per infection averted) with VCT and hospitalization having the largest incremental cost over VCT. It is the relative costs, not total cost of the strategy, that are most important. We see that while implementation of the intervention strategy ALL costs more, it is its incremental cost to VCT compared to the other strategies which matters.

**Figure 4:**
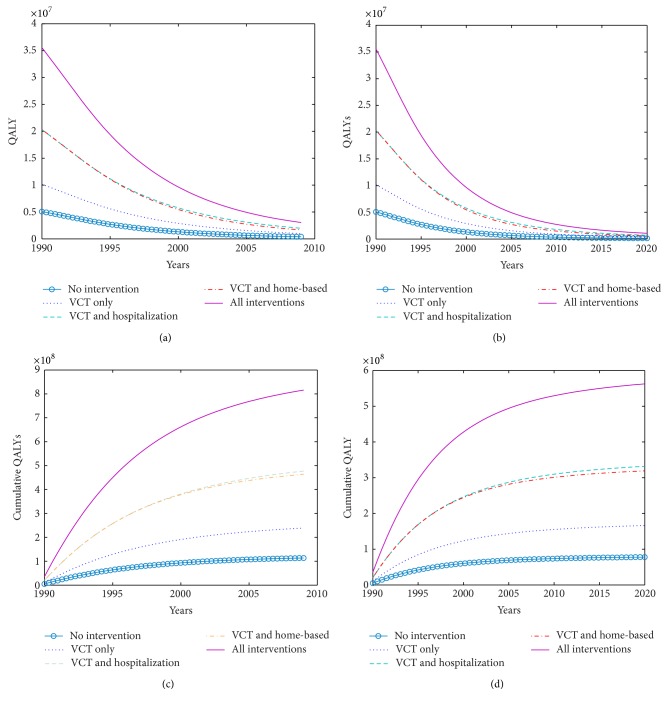
The trend of QALYs for each strategy per year for (a) 19 years and (b) 30 years. The discounted cumulative number of QALYs for 19 years and 30 years is shown in (c) and (d), respectively.

We again calculate the health benefits in terms of QALYs for each strategy. Discounted QALYs per year and cumulative QALYs for 19 years and 30 years are calculated.  Figure 4 shows the trend of the discounted QALYs per year and cumulative discounted QALYs for the various single or combined intervention strategies to control the HIV/AIDS epidemic. The discounted QALYs per year decrease as the epidemic is decreasing with time as shown in Figures 4(a) and 4(b). The cumulative discounted QALYs show that implementing strategy ALL brings in more health benefits, followed by VCT plus hospitalization with VCT giving the least number of QALYs. The cumulative discounted QALYs also decrease with time as shown in Figure 4(d). To determine the cost-effectiveness of the strategies, we calculated the ICERs taking VCT as the existing strategy.

**Figure 5:**
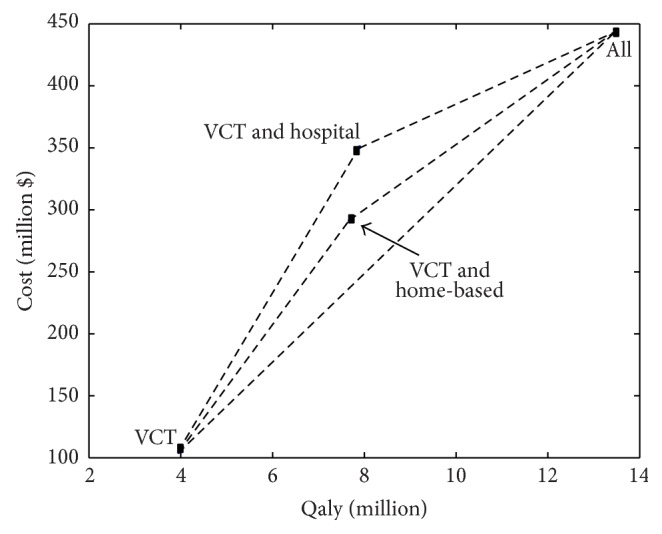
The cost and cost-effectiveness of the three strategies with VCT as the existing strategy.

Table 3 shows annual cost outcomes and the discounted QALYs per year for all the strategies. The ICER taking VCT as the existing strategy was calculated and this helps policy makers to decide whether to remain with VCT only or choose VCT with the other combinations. The ICER is the incremental cost per unit QALY gained. From Table 4, strategy ALL is the most cost-effective since its incremental cost is $35.40 per QALY gained followed by VCT plus CHBC and $50.75 per QALY gained with VCT plus hospitalization being the least cost-effective with $62.43. The discounted QALYs and the discounted costs for each strategy are represented by a point on the cost-effectiveness plane in Figure 5. It can be seen, from Figure 5, that all the strategies are potentially efficient being in the feasible plane of the cost-effectiveness plane where the strategies may be implemented. The ICER is represented in the cost-effectiveness graph by the slope of the lines joining the different strategies to VCT which is the existing strategy. From the graph in Figure 5, the line joining VCT to ALL has the lowest gradient followed by VCT to VCT plus CHBC with VCT plus hospitalization having the highest gradient. Therefore, strategy ALL is the most cost-effective strategy compared to the other two. This implies that implementing a combination of VCT, hospitalization, and CHBC gives better value for money. However, the decision to choose an efficient strategy from other efficient strategies depends on the maximum amount policy makers are willing to pay for the QALYs. The ranking of the interventions is *R*_*e*_ < *R*_*esh*_ < *R*_*esh*_*b*__ < *R*_*es*_ < *R*_0_, which slightly differ with the result, *R*_*e*_ < *R*_*esh*_*b*__ < *R*_*esh*_ < *R*_*es*_ < *R*_0_, which was obtained from the analysis of the reproduction numbers of the epidemiological model. While VCT plus hospitalization is more effective than VCT plus CHBC in the epidemiological model, it is not cost-effective. Therefore, interventions implemented concurrently are cost-effective as shown by the effective reproduction number *R*_*e*_ in the epidemiological model.

## 5. Sensitivity Analysis

We examine the sensitivity of the rankings of the strategies to the variation of some of the key parameters. We varied the proportion, *p*, of individuals who withdraw from risky sexual behaviours. Changes in risk behaviours have been shown to reduce HIV new infections [27]. We also varied the efficacy, *ϕ*, of home-based care since the introduction of home-based care is the novel part of the model. We also test the transmission contact rate, *β*, since all these strategies aim at reducing the number of infections which are all dependent on *β*. We varied all these parameters from 20% to 95%. We test the sensitivity of these parameters on the cost-savings and QALY-savings associated with the intervention strategies.

The results varying the proportion *p*, showing the costs and QALYs, are shown in Tables 4 and 5, respectively.

If more people withdraw from risky sexual behaviour, the costs for all the interventions are reduced. For example, if the proportion increases from 20% to 50% for the combined strategy, ALL, the costs are reduced by about 10.5%. The reduction will be about 13.7% if *p* changes from 20% to 95%. This implies that there is need for more educational campaigns to encourage people to refrain from risky sexual behaviour.

Health benefits from the strategies increase with an increase in the number of sexually active people practicing safe sex. The benefits increase by about 6.4% for a change in *p* from 20% to 95%.

The parameter *ϕ*, home-based care efficacy, only affects the interventions which include home-based care. The more efficient the home-based care programmes are, the less the expenses they incur. An increase of efficacy from 20% to 75% gives a reduction in cost of 1.7%. A reduction of 2.4% in costs is obtained for an increase from 20% to 95% in efficacy. Thus, there is an inverse relationship between the cost and the efficacy of CHBC. An increase in the efficacy of the community home-based care programs leads to an increase in the health benefits as shown in Table 6. In both VCT plus CHBC and ALL there is an increase in the number of discounted QALYs as the efficacy of the CHBC increases.

In Table 7, a decrease of the contact rate from 95% to 20% will result in a decrease in cost of 55.3%. On the other hand, if we begin with a situation, where the contact rate is low, an increase from 20% to 95% will result in an increase in cost of 123.6%. The cost is directly proportional to transmission contact rate. This shows that the transmission contact rate, *β*, has the greatest impact in the changes in both costs and health benefits accrued.

Health benefits, measured in discounted QALYs, have an inverse relationship with the transmission rate. In all the intervention strategies, a decrease in the contact rate results in an increase in the discounted QALYs. For example, for the strategy ALL, a reduction in contact rate from 95% to 20% will result in an increase in the discounted QALYs of 35.9% as shown in Table 8.

## 6. Conclusions

In this paper we evaluated the costs and benefits of HIV/AIDS intervention strategies using an interdisciplinary approach which weaves together the techniques of an epidemic transmission model, economics, and cost-effectiveness analysis. The intervention strategies considered are voluntary counselling and testing (VCT), VCT combined with hospitalization, VCT combined with community home-based care (CHBC), and finally VCT combined with hospitalization and CHBC (ALL). However, since VCT is the basis for all the intervention strategies suggested, this boils down to comparing CHBC and hospitalization singly or in combination. The interventions' costs and benefits were evaluated singly or in combination or when they are implemented concurrently. The main objectives were to find the most cost-effective strategy and compare the results with the epidemiological results in [16].

Our study results indicated that implementing all the strategies concurrently is the most cost-effective followed by VCT plus CHBC followed by VCT plus hospitalization. VCT only is the least cost-effective strategy. While VCT and H has slightly more health benefits and more effective in reducing the number of infections, its ICER is more than that of VCT and CHBC. Our results are also consistent with the epidemiological model results which also had more or less the same ranking of the strategies. They however differ in the fact that the VCT plus hospitalization is more effective in the epidemiological model but costly than VCT and CHBC. While the epidemiological model used the model reproduction number to determine the impact and ranking of the strategies, the economic model used the cost-effectiveness analysis concepts to determine the same results. In addition, the cost-effectiveness analysis used information from the epidemiological model. Data from the disease progression of the epidemiological model was used to determine the cost and benefits of the various strategies. This shows that the “marriage” of epidemiology and economics should be encouraged.

Our results also show that behaviour change, modelled by the parameters *p* and *m*, that accompanied the strategies, influences both the cost-effectiveness of an intervention strategy and dynamics of the epidemic. Both parameters are to do with behaviour as *m* indicates how individuals respond to the deaths of relatives, friends, and neighbours. This will then lead to the individual withdrawing from risky sexual activities, thus increasing the proportion *p* of the sexual active individuals withdrawing from risky sexual behaviour. The voluntary counselling and testing also lead individuals to reduce high risk behaviours. If there is no change of behaviour, our results show that the intervention strategies are more costly and reduce the number of QALYs whereas if there is behaviour change the costs are reduced and the QALYs increased. The transmission contact rate, *β*, is the most sensitive parameter which affects the epidemic size and the cost-effectiveness of the strategies. The results showed that a decrease of *β* from 95% to 20% will result in a decrease in cost by 55.3% and a corresponding increase in QALYS of 35.9% for the ALL strategy. The increase in effectiveness of CHBC measured by efficacy *ϕ* will also reduce the cost and increase the health benefits measured by the QALYs.

There are a number of limitations to this study. Firstly there are uncertainties in some of our assumptions on the variables and parameters. For example, we assumed that AIDS individuals discharged from hospitals are the only ones who go to community home-based care; yet it seems possible that the majority of AIDS individuals will access CHBC rather than going to the hospital. Secondly we assumed that an individual in CHBC is not admitted in the hospital. As a result of these assumptions our costs are bound to be affected since if more people living with HIV/AIDS (PLWHA) are admitted in hospitals this will increase the costs although it is likely to increase the QALYs. Thirdly our estimates on the costs involved in the home-based care programs need to be verified within the particular setting and this will require us to revisit our results which indicate that VCT and CHBC are more cost-effective than VCT and *H*. Lastly it is also important to note that data on risk sexual behaviour are not certain since they depend on self-reports which are difficult to verify. As pointed out in [22] the type of data may be related to age and this requires a further study. Decisions to implement a particular strategy are not only dependent on cost-effectiveness criteria but also dependent on other factors such as, what the policy maker is willing to pay and considers to be for money. Political commitment and infrastructure are some of the factors which must be taken into consideration. However, despite these limitations evidence on the cost-effectiveness presented in this study can help to inform decision makers which intervention strategies they can implement. As Klein et al. [33] noted, this study shows that interdisciplinary collaborations can help in improving the accuracy of predictions of the course and cost of the epidemic and help policy makers in implementing the correct strategies.

## Figures and Tables

**Figure 6:**
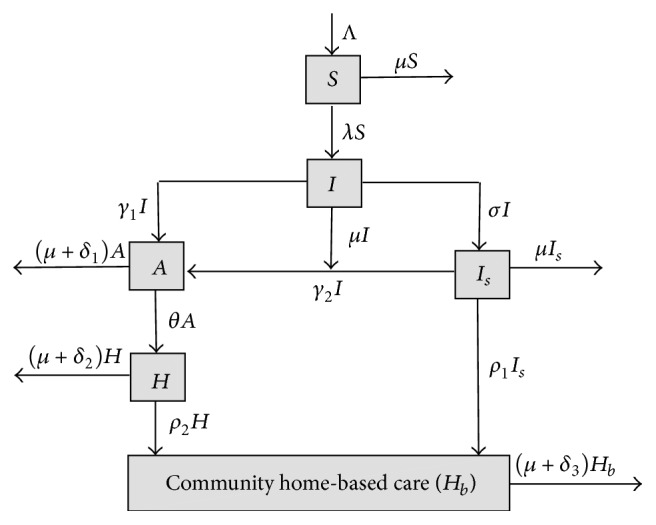
Model diagram showing movements of individuals between compartments.

**Figure 7:**
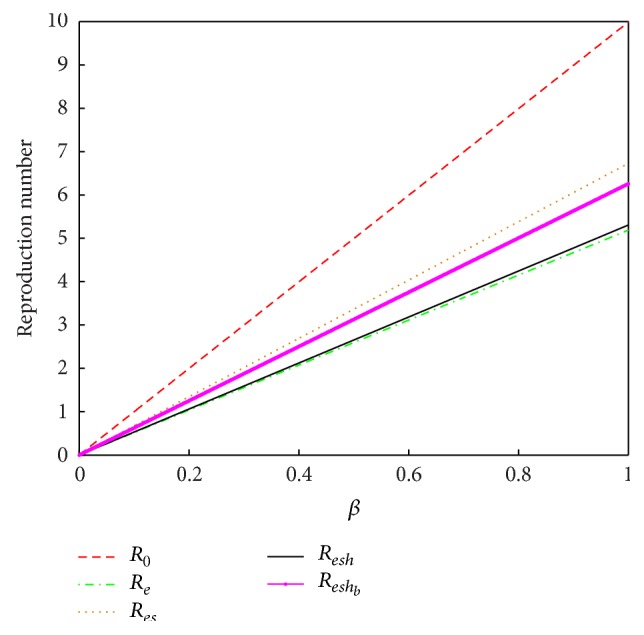
The relationship between the reproduction numbers as *β* changes. A comparison of the model reproduction numbers for the discussed cases showing the relationship between the various interventions as depicted by *R*_*e*_, *R*_*esh*_, *R*_*esh*_*b*__, *R*_*es*_, and *R*_0_.

**Table 1:** Parameter values used in the simulations.

Parameter	Symbol	Values	Source
Proportion of people using condoms	*τ*_*i*_(*i* = 1,2, 3,4, 5)	(0.55, 0.65, 0.75, 0.90, 0.85)	Estimate
Condom cost per person per year	*C*_*c*_	US$2.50	Estimate
Screening cost per person per year	*C*_*s*_	US$20	[19]
Hospital cost per person per year	*C*_*H*_	US$2000	Estimate
CHBC cost per person per year	*C*_*H*_*b*__	US$500	[20]
Running costs	*C*_*R*_	US$5000	Estimate
Discount rate	*r*	5%	[21]
Quality adjustment for a year of life in disease stage *i*	*q*_*i*_(*i* = 1,2, 3,4, 5)	(1, 0.83,0.42, 0.65, 0.171)	[22]

**Table 2:** Incremental cost-effectiveness ratio for the various combinations compared with VCT as the existing intervention strategy.

Intervention	Annual cost (US$ ×10^7^)	Infections averted ×10^4^	ICER $ ×10^3^/infection averted
VCT	1.0449	2.8517	

VCT + H	3.5058	2.9952	Dominated
VCT + CHBC	2.9138	3.2587	4.5919
ALL	4.4231	3.9141	2.302

**Table 3:** The cost and QALYs outcomes for the various combinations compared with VCT as the existing intervention strategy.

Intervention	Annual cost (US$ ×10^7^)	QALYs ×10^7^	ICER $/QALY
VCT only	1.0449	0.3955	

VCT + H	3.5058	0.7897	62.43
VCT + CHBC	2.9138	0.7678	50.75
ALL	4.4231	1.3499	35.40

**Table 4:** The annual cost outcomes for the various combinations when the proportion, *p*, of individuals withdraws from risky sexual behaviours.

Parameter *p* value	VCT only (US$ ×10^8^)	VCT + H (US$ ×10^8^)	VCT + CHBC (US$ ×10^8^)	ALL (US$ ×10^7^)
0.2	1.0464	3.6355	3.0105	4.5908
0.5	1.0449	3.5058	2.9138	4.4231
0.75	1.0413	3.3299	2.7867	4.2127
0.95	1.0324	3.0882	2.6238	3.9602

**Table 5:** The QALYs outcomes for the various combinations when the proportion, *p*, of individuals withdraws from risky sexual behaviours.

Parameter *p* value	VCT only (×10^7^)	VCT + H (×10^7^)	VCT + CHBC (×10^8^)	ALL (×10^7^)
0.2	0.3887	0.7759	0.7526	1.3259
0.5	0.3955	0.7897	0.7678	1.3499
0.75	0.4038	0.8063	0.7857	1.3779
0.95	0.4143	0.8268	0.8070	1.4108

**Table 6:** The QALYs outcomes for the various combinations when the proportion, *ϕ*, of individuals withdraws from risky sexual behaviours.

Parameter *ϕ* value	VCT only (×10^7^)	VCT + H (×10^7^)	VCT + CHBC (×10^8^)	ALL (×10^7^)
0.2	0.3955	0.7897	0.7669	1.3491
0.5	0.3955	0.7897	0.7679	1.3501
0.75	0.3955	0.7897	0.7689	1.3511
0.95	0.3955	0.7897	0.7697	1.3519

**Table 7:** The annual cost outcomes for the various combinations when the proportion, *β*, of individuals withdraws from risky sexual behaviours.

Parameter *β* value	VCT only (US$ ×10^8^)	VCT + H (US$ ×10^8^)	VCT + CHBC (US$ ×10^8^)	ALL (US$ ×10^7^)
0.2	0.9931	1.9284	1.5838	2.0848
0.5	1.0371	2.9733	4.4729	3.6010
0.75	1.0446	3.4600	2.8779	4.3561
0.95	1.0459	3.6736	3.0422	4.6614

**Table 8:** The QALYs outcomes for the various combinations when the proportion, *β*, of individuals withdraws from risky sexual behaviours.

Parameter *β* value	VCT only (×10^7^)	VCT + H (×10^7^)	VCT + CHBC (×10^8^)	ALL (×10^7^)
0.2	0.5091	1.0187	1.0138	1.7715
0.5	0.4316	0.8634	0.8488	1.4863
0.75	0.3987	0.7963	0.77518	1.3622
0.95	0.3833	0.7647	0.7405	1.3039

**Table 9:** Variables, parameters, and parameter values used in the model.

Variable	Description	
*S*(*t*)	Susceptible individuals	
*I*(*t*)	Unidentified asymptomatic HIV infected individuals	
*I*_*s*_(*t*)	Screened and identified HIV infected individuals	
*A*(*t*)	Symptomatic with full blown AIDS individuals	
*H*	Hospitalized AIDS	
*H*_*b*_	Community home-based care	

Parameter	Description	Range/source

Λ	Recruitment rate into susceptible class	52600
*β*	The transmission contact rate	(0, 1) [28]
*m*	Measuring how individuals respond to the increase or decrease of mortality due to HIV/AIDS	(10, 100)—Fitted
*μ*	Non-HIV/AIDS death rate	0.029 [20]
*ϕ*	CHBC efficacy rate	(0, 1)—Fitted
*p*	Withdrawal proportion from risky sexual activities due to counselling	(0, 1)—fitted
*η*_1_, *η*_2_, *η*_3_	Adjustment factors due to behavioural change	(0, 3)—fitted
*ρ*_1_	Rate of seeking care and treatment from CHBC by the screened infective individuals	(0.01, 0.053)—fitted
*ρ*_2_	Rate of seeking care and treatment from CHBC of AIDS individuals discharged from hospital	0.053—[24]
*θ*	Recruitment rate into hospital	(0.05, 0.8)—[29]
*γ*_1_	Progression rate to AIDS of unidentified infective individuals	(0.1,0.2)—[5]
*γ*_2_	Progression rate to AIDS of screened infective individuals	1/12—[20]
*σ*	Screening rate of unidentified infective individuals	(0.05, 0.3)/[13]
*δ*_*i*_ (*i* = 1,2, 3)	Disease induced death rates	(0.05, 0.33)—[30–32]

## Appendices

### A. Variables and Parameters

See Table 9.

### B. Model Flow Diagram and Equations

The dynamical system described by Figure 6 is given by the following set of differential equations:

(B.1) S˙=Λ−λS−μS,I˙=λS−(μ+σ+γ1)I,I˙s=σI−(μ+γ2+ρ1)Is,A˙=γ1I+γ2Is−(μ+θ+δ1)A,H˙=θA−(μ+ρ2+δ2)H,H˙b=ρ1Is+ρ2H−(μ+δ3)Hb,

where *λ* = *βe* ^−*m*(*δ*_1_*A*+*δ*_2_*H*+*δ*_3_*H*_*b*_)^((*I* + (1 − *p*)(*η*_1_ *I*_*s*_ + *η*_2_ *A* + *η*_3_(1 − *ϕ*)*H*_*b*_))/*N*), *η*_1_, *η*_2_, and *η*_3_ measure the relative infectivity of *I*_*s*_, *A*, and *H*_*b*_, when compared to *I*, and *p* is a fraction of individuals who have been screened, developed AIDS, and in the CHBC who withdrew from any sexual activity.

### C. Comparison of Reproduction Numbers

A comparison of the model reproduction numbers for the various cases is shown in Figure 7.

The relationship between the various interventions is given in terms of the subreproduction numbers with the following relations:

(C.1) $$\begin{array}{c} R_{e}<R_{esh}<R_{esh_{b}}<R_{es}<R_{0}. \end{array}$$
